# Glucose homeostasis and insulin resistance: prevalence, gender differences and predictors in adolescents

**DOI:** 10.1186/1758-5996-6-100

**Published:** 2014-09-16

**Authors:** Irena Aldhoon-Hainerová, Hana Zamrazilová, Lenka Dušátková, Barbora Sedláčková, Petr Hlavatý, Martin Hill, Richard Hampl, Marie Kunešová, Vojtěch Hainer

**Affiliations:** Institute of Endocrinology, Obesity Management Center, Národní 8, 116 94 Prague 1, Czech Republic; Department of Pediatrics and Center for Research of Diabetes, Metabolism and Nutrition, Third Faculty of Medicine, Charles University, Šrobárova 50, 100 34 Prague 10, Czech Republic; Faculty of Science, Charles University, Albertov 6, 128 43 Prague 2, Czech Republic

**Keywords:** Adolescence, Glucose homeostasis, HOMA-IR prediction, Insulin resistance, Metabolic syndrome, Obesity, Type 2 diabetes

## Abstract

**Background:**

Adolescence, due to transient pubertal insulin resistance (IR), is associated with a higher risk for disturbances of glucose metabolism. The aim of our study was 1) to investigate the prevalence of disturbances of glucose metabolism, 2) to define gender specific homeostasis model assessment of insulin resistance (HOMA-IR) thresholds associated with increased cardiometabolic risks and 3) to provide predictors of HOMA-IR.

**Methods:**

The studied cohort consisted of Czech adolescents aged 13.0-17.9 years: 1,518 individuals of general population and three studied groups according weight category (615 normal weight, 230 overweight and 683 obese). The prevalence of IR, impaired fasting glucose (IFG) and type 2 diabetes was assessed. Risky HOMA-IR thresholds based on components of metabolic syndrome were investigated. HOMA-IR prediction was calculated taking into account age, blood pressure, multiple anthropometric, biochemical and hormonal parameters.

**Results:**

In general population cohort, the prevalence of IFG and type 2 diabetes was 7.0% and <0.5%, respectively. Boys regardless of weight presented significantly higher levels of blood glucose and higher prevalence of IFG than girls. Obese boys were found more insulin resistant than obese girls. HOMA-IR thresholds of 3.6 for girls and 4.4 for boys were associated with increased cardiometabolic risks. For both genders, the model of HOMA-IR prediction was composed of age, BMI, ratio of free triiodthyronine to free thyroxine, gamma-glutamyltransferase activity and levels of triglycerides and sex hormone-binding globulin.

**Conclusions:**

The type 2 diabetes in adolescents, including those who were obese, was rarely diagnosed. Obese adolescent boys were at greater risk for IR and for IFG than obese girls. In adolescence, thresholds of HOMA-IR in contrast to predictors were found gender specific.

## Introduction

Insulin resistance (IR) together with an impaired insulin secretion does play a role in the pathogenesis of type 2 diabetes [1]. A degree of IR is influenced by age, gender, race and ethnicity, a stage of sexual development, total adiposity and fat distribution [2–4]. To date, there is no universally accepted pediatric definition for IR. The homeostasis model assessment of insulin resistance (HOMA-IR) is simple method to measure IR [5] and even in obese children strongly correlates with the results derived from clamp studies [6]. An identification of insulin resistant adolescent is highly important as the occurrence of type 2 diabetes coincides with the peak of pubertal IR [7]. The interpretation of HOMA-IR value is particularly challenging at adolescence. Several HOMA-IR thresholds to define IR in adolescence have been suggested [8–11].

Fasting insulin level is sometimes used as a marker of IR. A study of Moran et al. demonstrated a significant correlation between glucose uptake during insulin clamp and fasting insulin levels [4]. IR, particularly in girls, has been shown to be associated with a decreased ratio of C-peptide to insulin [12].

Patients with impaired fasting glucose (IFG) are referred to as having prediabetes and they are considered at risk for future development of type 2 diabetes [13]. Data from National Health and Nutrition Examination Survey showed that the prevalence of IFG in United States (US) adolescents was 7.0% [14]. Higher prevalence of IFG is usually found in obese adolescents than in normal weight counterparts [14]. The prevalence of type 2 diabetes in children varies across the world but an increasing trend due to obesity epidemic is observed. A higher prevalence of type 2 diabetes is found in the US children mainly in those of non-White European descent [15]. In the Northern and Central Europe type 2 diabetes is still, even in obese children, rarely diagnosed and its prevalence ranges 0.5 to 1.5% [16–18].

Metabolic syndrome due to IR predisposes affected individuals to type 2 diabetes. Tresaco et al. showed that HOMA-IR is a useful tool to detect children and adolescents with this condition [8]. Several studies investigated predictors of pubertal IR e.g. anthropometric parameters [19], body composition [20], adipokines [20–22] and several hormones [23, 24].

The first aim of our study was to reveal the prevalence of IR, IFG and type 2 diabetes in normal weight, overweight and obese Czech adolescents, and in a general population cohort. Second, we aimed to investigate the effects of gender and weight status on levels of blood glucose, insulin, C-peptide, C-peptide to insulin ratio and HOMA-IR. Our third aim was to examine the level of HOMA-IR in relation to the presence of parameters defining metabolic syndrome. Finally, we intended to assess major predictors of HOMA-IR from the whole cohort of studied Czech adolescents.

## Methods

### Study population

The cohort for the present study was constituted of Czech adolescents aged 13.0-17.9 years from the Childhood Obesity Prevalence And Treatment project which consists of a general population cohort (all body weight categories) and of in- and out-patient weight intervention cohort (overweight and obese individuals only). Analyses were performed in 1,518 Czech adolescents of general population (775 girls, 743 boys; 23.9% of girls and 29.3% of boys were either overweight or obese) and in three weight categories: 615 normal weight (322 girls and 293 boys) for Body Mass Index (BMI) 25.–75. percentile (derived from the general population cohort only), 230 overweight (116 girls, 114 boys) for BMI 90.–97. percentile and 683 obese (381 girls, 302 boys) for BMI ≥ 97. percentile for sex and age of the Czech reference [25]. The overweight and obese groups were constituted from both the general population cohort and from the intervention study of the above mentioned project. A detailed design of the project has already been described [26].

### Ethics statement

The study protocol was approved by the Ethical Committee of the Institute of Endocrinology in Prague and was in accordance with the Helsinki declaration II. All participants and their parent(s)/guardian(s) signed an informed consent before the initiation of study procedures.

### Clinical examination, anthropometry and body composition

Following parameters were investigated: age; systolic and diastolic blood pressure; anthropometric parameters and indexes {weight, height, waist circumference, abdominal circumference, hip circumference, arm circumference and theirs z scores; sagittal abdominal diameter; skinfolds: suprailiac, biceps, triceps, abdomen and subscapular; fat assessed by bioimpedance (Tanita BC-418 MA, Tanita AB 140 MA Viscan, Tanita Corporation, Tokyo, Japan): trunk fat, total body fat, and visceral fat; indexes: BMI, BMI z-score, waist-to-hip ratio, waist-to-height ratio, body adiposity index: [(hip circumference)/((height)1.5] - 18, a body shape index: waist circumference divided by BMI2/3 height1/2}.

### Biochemical and hormonal parameters

We investigated following biochemical parameters: blood glucose, total cholesterol, high-density lipoprotein cholesterol, low-density lipoprotein cholesterol, triglyceride (TG), C-peptide, C-reactive protein, uric acid, activity of alanine aminotransferase, aspartate aminotransferase and gamma-glutamyl aminotransferase (GMT) and the following hormonal parameters: insulin, free triiodothyronine (fT3), free thyroxine (fT4), thyroid stimulating hormone, prolactin, cortisol, dehydroepiandrosterone sulfate, sex hormone-binding globulin (SHBG), testosterone, luteinizing hormone, follicle stimulating hormone, estradiol, progesterone, adiponectin, adipsin, ghrelin, glucose insulinotropic peptide, glucagon-like-peptide 1, glucagon, leptin, plasminogen activator inhibitor-1 (PAI-1), resistin and visfatin. Ratio of fT3 to fT4 was calculated.

Anthropometric procedures, laboratory assays and evaluations have been described in a previously published paper [26].

### Definitions of glucose homeostasis disturbances, IR and metabolic syndrome

According to the American Diabetes Association IFG is defined by fasting blood glucose 5.6–6.9 mmol/l and type 2 diabetes by fasting blood glucose ≥ 7.0 mmol/l [13]. IR was evaluated by the HOMA-IR that was calculated by the following formula: fasting plasma insulin (microunits per liter) × fasting glucose (millimoles per liter)/22.5 [5]. In order to estimate IR in our cohorts, we used the thresholds of HOMA-IR > 2.5 and > 4.0 [5, 27]. HOMA-IR ≤ 4.0 was shown to be associated with a little probability of type 2 diabetes development [28]. Parameters of the International Diabetes Federation (IDF) definition of the metabolic syndrome were used in our analyses [29].

### Statistical analyses

To eliminate skewed data distribution and heteroscedasticity, the original data were transformed to a Gaussian distribution by a Box-Cox transformation before further processing using the statistical software Statgraphics Centurion, version XV from Statpoint Inc. (Herndon, Virginia, USA). Data were analyzed using non-parametric tests: Mann–Whitney test (for differences between two independent groups) and Kruskal-Wallis multiple comparisons (for differences among three or more independent groups). Categorical data were tested by Chi-square test. The statistical software NCSS 2004 (Kaysville, Utah, 190 USA) were used. P-value (two-tailed) < 0.05 was considered statistically significant.

In order to detect relationships between HOMA-IR and studied parameters we applied a multivariate regression with reduction of dimensionality, known as OPLS [30]. All subjects that had been assessed for the present study were used for the prediction of HOMA-IR. Analyses were performed separately for boys and girls. OPLS allowed us to find a model predicting HOMA-IR based both on the minimum number of widely available parameters but also on the best predictability. We tested the relevance of individual variables for the model using a criterion variable importance. At the first stage, all above mentioned parameters, excluding levels of blood glucose and insulin, were studied for HOMA-IR prediction. We constructed two models for HOMA-IR prediction. The first model comprised components with the highest predictability. The second model was based on the first model and aimed at components that are widely available in clinical practice (clinical model).

The statistical software SIMCA-P + Version 12.0.0.0 from Umetrics AB (Umeå, Sweden) was used for data analyses. The software enabled us to find the number of the relevant components utilizing the prediction error sum of squares and also allowed the detection of multivariate non-homogeneities and testing the multivariate normal distribution and homoscedasticity.

## Results

### Disturbances of glucose metabolism and IR

Basic characteristics and only those parameters, which were found as major predictors of HOMA-IR, for all studied groups are presented in Table 1. Between girls and boys of each studied group, there were no differences in age and BMI z-score except for BMI z-score in the overweight cohort (Table 1). Table 1 also shows the prevalence of IFG, type 2 diabetes and of IR defined by two thresholds of HOMA-IR in each cohort. IFG was more common in boys than in girls regardless of weight status (Table 1).

**Table 1:** **Baseline characteristics of studied cohorts – general population, normal weight, overweight and obese**

Parameter	General population			Normal weight			Overweight			Obese		
	Girls (n = 775)	Boys (n = 743)	p	Girls (n = 322)	Boys (n = 293)	p	Girls (n = 116)	Boys (n = 114)	p	Girls (n = 381)	Boys (n = 302)	p
Age* **(years)**	16.02 (15.10;17.00)	16.08 (15.13;17.00)	0.745	16.12 (15.16; 17.02)	15.87 (15.08; 16.84)	0.097	15.66 (14.82; 16.92)	16.04 (14.46; 17.08)	0.465	15.18 (14.07; 16.42)	15.21 (14.03;16.56)	0.990
BMI* **(kg/m** ^**2**^ **)**	21.25 (19.41; 23.61)	21.47 (19.64; 24.48)	0.022	20.28 (19.58; 21.15)	20.29 (19.58; 21.08)	0.963	24.79 (24.17; 25.48)	24.75 (24.08; 25.70)	0.934	29.95 (27.89; 33.07)	30.34 (28.13; 33.95)	0.146
BMI* **z-score**	0.24 (-0.46; 1.15)	0.22 (-0.39; 1.25)	0.280	-0.12 (-0.41; 0.19)	-0.15 (-0.39; 0.10)	0.114	1.60 (1.39; 1.60)	1.43 (1.22; 1.66)	0.000	3.53 (2.78; 4.65)	3.50 (2.69; 4.72)	0.506
FBG* **(mmol/l)**	4.83 (4.55; 5.09)	5.09 (4.84; 5.35)	0.000	4.79 (4.52; 5.06)	5.08 (4.82; 5.31)	0.000	4.79 (4.52; 5.07)	5.13 (4.90; 5.43)	0.000	4.82 (4.52; 5.14)	5.08 (4.81; 5.34)	0.000
Insulin* **(mIU/l)**	10.57 (7.91; 14.08)	9.78 (7.25; 13.70)	0.010	9.80 (7.6; 12.17)	8.4 (6.42; 11.61)	0.001	11.84 (9.24; 15.16)	11.30 (8.85; 15.29)	0.626	13.93 (9.72; 19.84)	15.98 (11.22; 24.83)	0.001
C-peptide* **(nmol/l)**	0.75 (0.63; 0.89)	0.69 (0.57; 0.86)	0.000	0.71 (0.60; 0.82)	0.64 (0.53; 0.77)	0.000	0.79 (0.68; 0.91)	0.78 (0.65; 0.92)	0.723	0.92 (0.74; 1.13)	0.94 (0.77; 1.23)	0.040
HOMA-IR*	2.26 (1.66; 3.11)	2.23 (1.63; 3.20)	0.681	2.09 (1.54; 2.72)	1.89 (1.43; 2.71)	0.099	2.52 (1.92; 3.32)	2.65 (1.91; 3.65)	0.360	2.95 (2.10; 4.29)	3.56 (2.51; 5.74)	0.000
C-peptide/Insulin*	10.09 (8.37; 11.93)	9.96 (8.48; 12.09)	0.543	10.39 (9.15; 12.13)	10.47 (8.97; 12.41)	0.728	9.50 (8.16; 11.13)	9.52 (7.99; 11.24)	0.814	9.21 (7.73; 11.15)	8.54 (6.98; 10.08)	0.000
TG* **(mmol/l)**	0.87 (0.67; 1.18)	0.82 (0.63; 1.08)	0.002	0.83 (0.65; 1.19)	0.74 (0.58; 0.92)	0.000	0.90 (0.66; 1.18)	0.94 (0.66; 1.23)	0.714	0.96 (0.74; 1.38)	1.12 (0.82; 1.64)	0.000
GMT* **(μkat/l)**	0.20 (0.17; 0.25)	0.26 (0.22; 0.337)	0.000	0.19 (0.16; 0.24)	0.25 (0.21; 0.30)	0.000	0.22 (0.18; 0.30)	0.30 (0.25; 0.42)	0.000	0.25 (0.2; 0.32)	0.34 (0.27; 0.51)	0.000
fT3/fT4*	0.35 (0.32; 0.40)	0.39 (0.35; 0.44)	0.000	0.35 (0.32; 0.40)	0.39 (0.35; 0.44)	0.000	0.36 (0.32; 0.39)	0.40 (0.36; 0.46)	0.000	0.35 (0.31;0.40)	0.40 (0.36; 0.46)	0.000
SHBG* **(nmol/l)**	57.77 (37.35; 82.62)	31.63 (23.15; 43.80)	0.000	63.04 (45.26; 89.82)	35.45 (25.94; 48.58)	0.000	44.50 (27.99;71.62)	29.49 (21.76; 43.38)	0.000	31.90 (22.59; 45.54)	22.62 (15.76; 43.78)	0.000
Testosterone* **(nmol/l)**	0.30 (0.22; 0.40)	5.25 (4.02; 6.73)	0.000	0.30 (0.22; 0.41)	5.97 (4.72; 7.10)	0.000	0.31 (0.23; 0.43)	4.60 (3.32; 6.41)	0.000	0.36 (0.26; 0.48)	2.96 (1.75; 4.34)	0.000
Adiponectin* **(mg/l)**	7.82 (5.36; 10.56)	6.00 (4.55; 8.17)	0.000	8.61 (6.09; 11.04)	6.59 (5.05; 8.43)	0.000	6.34 (4.86; 8.98)	5.28 (4.00; 7.42)	0.001	5.58 (3.96; 7.81)	4.52 (3.30; 6.00)	0.000
Ghrelin* **(pg/ml)**	669.11 (503.72; 899.49)	600.59 (459.78; 829.32)	0.000	688.97 (507.15; 918.92)	597.65 (460.22; 869.06)	0.006	646.55 (520.87; 862.62)	580.91 (480.43; 783.88)	0.138	582.61 (445.71; 759.52)	543.17 (406.74; 686.29)	0.012
Leptin* **(ng/ml)**	4.81 (3.07; 7.29)	0.89 (0.51; 2.18)	0.000	4.02 (2.81; 5.81)	0.63 (0.41; 0.89)	0.000	7.25 (5.27; 9.96)	2.16 (1.21; 3.20)	0.000	9.41 (6.20; 14.69)	5.48 (3.28; 8.32)	0.000
PAI-1* **(ng/ml)**	2.97 (2.04; 3.94)	3.03 (2.35; 4.10)	0.002	2.64 (1.89; 3.43)	2.79 (2.21; 3.57)	0.033	3.46 (2.45; 4.70)	3.37 (2.74; 4.45)	0.842	4.40 (3.19; 6.19)	4.87 (3.53; 6.76)	0.013
**HOMA-IR > 2.5 (n,%)**	314 (40.50%)	304 (40.90%)	0.874^†^	98 (30.40%)	82 (28.00%)	0.505^†^	58 (50.00%)	59 (51.80%)	0.790^†^	251 (65.90%)	228 (75.50%)	0.006^†^
**HOMA-IR > 4 (n,%)**	84 (10.80%)	106 (14.30%)	0.044^†^	15 (4.70%)	21 (7.20%)	0.186^†^	13 (11.20%)	20 (17.50%)	0.170^†^	111 (29.10%)	133 (44.00%)	0.000^†^
**IFG (n,%)**	34 (4.40%)	72 (9.70%)	0.000^†^	12 (3.70%)	20 (6.80%)	0.084^†^	2 (1.70%)	15 (13.20%)	0.001^†^	22 (5.80%)	29 (9.60%)	0.060^†^
**T2DM (n,%)**	1 (0.13%)	2 (0.27)	‡	0	0	---	0	0	---	2 (0.50%)	1 (0.30%)	‡

BMI, body mass index; T2DM, type 2 diabetes; FBG, fasting blood glucose; fT3/fT4, ratio of free triiodothyronine to free thyroxine; GMT, gamma-glutamyltransferase; HOMA-IR, homeostasis model assessment of insulin resistance; IFG, impaired fasting glucose; PAI-1, plasminogen activator inhibitor-1; TG, triglycerides. *Data are described as median, lower and upper quartiles. ^†^Chi-test was used. ^‡^Not calculated due to small number of probands. Data significant at p < 0.05.

Significant differences in BMI z-scores, levels of insulin, C-peptide, C-peptide to insulin ratio (except for the ratio in overweight vs. obese girls) and HOMA-IR (p = 0.000) but not in blood glucose level were found when weight categories were compared between each other (normal weight vs. overweight; normal weight vs. obese; overweight vs. obese) in analyses within each gender (data not shown). In all cohorts, boys presented significantly greater level of blood glucose than girls (p = 0.000) (Table 1). Levels of insulin and C-peptide were significantly higher in girls than in boys in the normal weight and in the general population cohorts (p = 0.001) (Table 1). However, obese boys had significantly higher insulin, C-peptide and HOMA-IR than obese girls (Table 1). C-peptide to insulin ratio gradually decreased with an increasing body weight. No gender differences in this ratio were noted except for obese individuals (Table 1).

### HOMA-IR thresholds associated with cardiometabolic risks

In both genders, HOMA-IR gradually increased with increasing number of parameters derived from metabolic syndrome definition. HOMA-IR of 3.6 for girls and of 4.4 for boys were identified as cut-off values that corresponded to increased cardiometabolic risks defined as a presence of three components of metabolic syndrome (Table 2). These HOMA-IR values represent the 84.0 and 87.0 percentile in girls and boys of general population, respectively.

**Table 2:** **HOMA-IR values in relation to the number of metabolic syndrome components**

Number of metabolic syndrome components (number of girls/boys)	HOMA-IR	
	Girls	Boys
0 (468/301 girls/boys)	2.12 (1.53; 2.84)	1.90 (1.42; 2.61)
1 ( 381/359 girls/boys)	2.55 (1.80; 3.45)	2.28 (1.65; 3.11)
2 (174/168 girls/boys)	2.85 (2.09; 4.37)	3.19 (2.20; 5.03)
3 (54/76 girls/boys)	3.60 (2.56; 5.69)	4.44 (2.88; 6.25)
4 (4/29 girls/boys)	5.86 (4.90; 9.19)	6.38 (4.26; 7.98)
5 (0/3 girls/boys)	---	7.19 (4.33; 13.93)

HOMA-IR, homeostasis model assessment of insulin resistance. Data are described as median, lower and upper quartiles.

### HOMA-IR prediction

**Figure 1:**
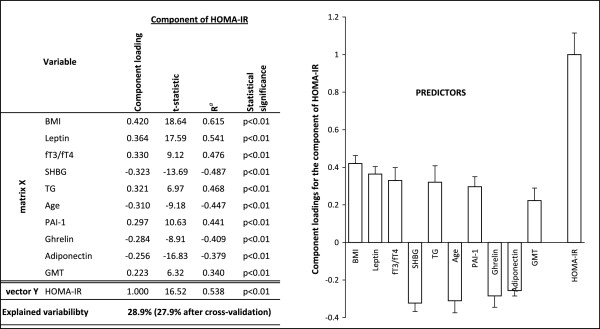
**Relationships between HOMA-IR (vector Y) and major predictors (matrix X) for girls.** Ra is a component loading expressed as a correlation coefficient with the predictive component. T-statistic is a ratio of component loading and statistical error. BMI, body mass index; fT3/fT4, ratio of free triiodothyronine to free thyroxine; GMT, gamma-glutamyl aminotransferase; HOMA-IR, homeostasis model assessment of insulin resistance; PAI-1, plasminogen activator inhibitor-1; SHBG, sex hormone-binding globulin; TG, triglycerides.

**Figure 2:**
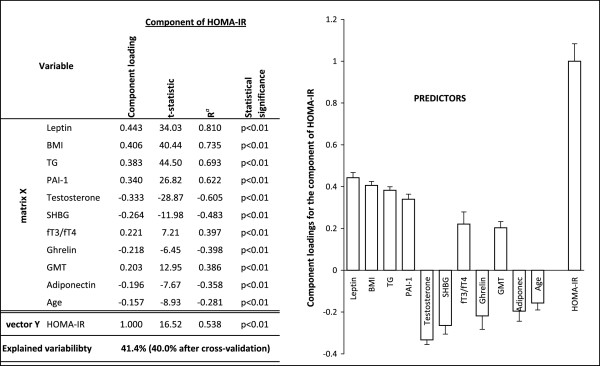
**Relationships between HOMA-IR (vector Y) and major predictors (matrix X) for boys.** Ra is a component loading expressed as a correlation coefficient with the predictive component. T-statistic is a ratio of component loading and statistical error. BMI, body mass index; fT3/fT4, ratio of free triiodothyronine to free thyroxine; GMT, gamma-glutamyl aminotransferase; HOMA-IR, homeostasis model assessment of insulin resistance; PAI-1, plasminogen activator inhibitor-1; SHBG, sex hormone-binding globulin; TG, triglycerides.

Out of 68 analyzed parameters, 57 parameters in boys (43.2% variability of the dependent variable explained by OPLS method) and 51 parameters in girls (30.1% variability of the dependent variable explained by OPLS method) showed a significance to HOMA-IR prediction. At this stage, we first selected those parameters that presented with the highest prediction and were more or less shared by both genders. Similar prediction of most of the studied anthropometric parameters were found, thus, for the further model analyses we only included BMI. In the predictive model, age, BMI, ratio of fT3 to fT4, GMT activity, levels of TG, SHBG, leptin, PAI-1, ghrelin, adiponectin and testosterone explained 41.4% of the HOMA-IR in boys. Except for the level of testosterone, girls presented with the same predictors but the HOMA-IR variability was explained by 28.9% (Figures 1 and 2). Construction of the second predictive model was based on inclusion of the minimum of widely used parameters in clinical practice such as age, BMI, ratio of fT3 to fT4, GMT activity, levels of TG and SHBG. The model showed still satisfactory predictability (37.8% variability of the dependent variable explained by OPLS method in boys, resp. 20.5% in girls).

## Discussion

### Disturbances of glucose metabolism and IR

Our study of Czech adolescents presents an overview of glucose metabolism with respect to different weight categories and to gender. To date, there are no data on glucose homeostasis disturbances in Czech adolescents. In the representative cohort of Czech adolescents, IFG was found in 7.0% with a higher prevalence in boys than in girls (9.7 vs. 4.4%). This finding is in accordance with US data which showed similar overall prevalence of IFG in adolescents and also higher prevalence in boys than in girls (10.0% vs. 4.0%) [14]. We further showed that boys are at greater risk for IFG regardless weight status. Our results of an overall prevalence of type 2 diabetes in general population (0.2%) and obese adolescents (0.4%) indicate that type 2 diabetes is still a rare diagnosis even in the population of obese Czech adolescents. It is known that the prevalence varies by ethnicity and that much greater prevalence of type 2 diabetes is found in the US non-White European descent [15]. Lower prevalence of type 2 diabetes may partly be explained by ethnically homogenous Czech Caucasian population. In contrast to results from US it has been demonstrated that type 2 diabetes in the Northern and Central Europe is still rarely diagnosed even in obese children [16]. It is highly interesting that the level of fasting blood glucose was similar across weight categories but always significantly higher in boys than in girls. Brandou et al. found that the level of blood glucose was significantly higher in obese than in normal weight children but only in prepubertal period [31]. Several studies concluded that men had tendency towards higher prevalence of abnormalities of glucose homeostasis than women [32, 33]. A recent study performed on Chinese children and adolescents has also reported significantly higher levels of fasting blood glucose in boys in comparison to girls regardless of weight status [20]. Our results as conclusions of others indicate that the HOMA-IR cut-off point of 2.5 is not suitable for adolescence [9]. Interestingly, only in the obese group there was a significant gender difference in the prevalence of IR. This result emphasizes the fact that obese adolescent boys are at greater risk for IR than girls of comparable degree of obesity.

It has already been demonstrated by several investigators that gender has an effect on insulin sensitivity [1, 3, 4, 9, 34]. In normal weight Czech adolescents as well as in the general population cohort, levels of insulin and C-peptide were found significantly higher in girls than in boys. This finding would therefore confirm the fact than at puberty girls tend to be more insulin resistant than boys. Several studies showed a higher IR in girls than in boys even after adjustment for weight status, several anthropometric parameters, race and ethnicity [1, 4]. According to our results, obesity in males led to a significantly higher levels of insulin, C-peptide, HOMA-IR and lower C-peptide to insulin ratio than in obese girls. This is in line with conclusion of Kurtoglu et al. who demonstrated that HOMA-IR cut-off values in the pubertal stage were significantly higher in boys than in girls [10].

### HOMA-IR thresholds associated with cardiometabolic risks

HOMA-IR was shown to be a useful tool to detect children and adolescents with metabolic syndrome [8]. As expected and in accordance with the study of Chinese children and teenagers we confirmed the trend of increasing HOMA-IR values with the increasing number of metabolic syndrome components [35]. At adolescence, HOMA-IR thresholds of 3.6 for girls and 4.4 for boys were associated with increased cardiometabolic risks. In the agreement with the conclusion of Kurtoglu et al., we also emphasize the necessity of gender specific cut-off thresholds [10].

### HOMA-IR prediction

According to our findings, a broad variety of parameters had a predictive power for HOMA-IR. As expected the majority of studied anthropometric parameters played a role in the index prediction. Due to the fact that the prediction rate among anthropometric parameters was similar, only BMI was included for further model assessment. BMI is widely used in clinical practice, in epidemiological studies and according to our findings had a comparable prediction to other anthropometric parameters, e.g. waist circumference. A study by Bosy-Westphal et al. concluded that waist circumference and BMI have an equivalent value for obesity-related metabolic risk assessment [36]. The same study also showed that measurement of body fat mass has no advantage over BMI and waist circumference in the prediction of obesity-related metabolic risk [36]. We are aware of the fact that in normal weight but insulin resistant subjects other factors than BMI probably play a role in the development of IR, e.g. fat distribution, family history. According to our predictive models, age in girls seemed to be stronger predictor of HOMA-IR than in boys. Younger age particularly in girls was associated with higher HOMA-IR. This is probably due to the fact that younger girls of our cohort were at the peak of IR associated with puberty.

In our cohort, GMT activity and levels of TG, SHBG, leptin, PAI-1, ghrelin and adiponectin in both genders and testosterone in boys were revealed as key components in HOMA-IR prediction. A strong correlation between IR and TG has already been confirmed by several studies [37]. Significant associations between IR and non-alcoholic fatty liver disease as well as similar risk and protective factors for these two inter-related disorders are known [38, 39]. It is well recognized that GMT activity is a marker of oxidative stress and is associated with an increased risk of cardiovascular disease and components of metabolic syndrome [40].

According to our results, testosterone was associated with HOMA-IR prediction in boys. Testosterone is usually decreased in obese males than in their lean counterparts. It has also been shown that glucose disposal was significantly associated with serum testosterone, even after controlling for BMI and Tanner stage and thus, linking the presence of IR with hypogonadism [41]. The level of SHBG was found as an important predictor of IR in both genders. A strong correlation between level of leptin and IR measured by HOMA-IR even after adjustment for gender and BMI has previously been shown [42]. In a recently published study, leptin was also found as a strong and independent predictor of HOMA-IR in boys and in girls [20]. However, some authors indicated this relationship due to increased fat mass [43]. PAI-1 represents an independent risk factor for cardiovascular diseases and its level and activity is increased in type 2 diabetics [44]. A study on young adults demonstrated that PAI-1 concentrations were higher in subjects with impaired glucose tolerance than in subjects with normal glucose tolerance even having comparable age, BMI, waist circumference and fat mass [44]. In relation to HOMA-IR, ghrelin was another parameter with a predictive power in Czech girls and boys. Bacha et al. suggested that ghrelin suppression might be another feature of IR in overweight adolescents [23]. A correlation of adiponectin with IR has already been demonstrated [21, 22].

In the analyses, in which all studied parameters had been taken into account, TSH in both genders, fT4 in boys and fT3 in girls were found to have some predictive power for HOMA-IR. However, the ratio of fT3 to fT4 had stronger prediction than the above mentioned parameters, thus we included this ratio in the predictive models. Several studies demonstrated that a higher fT3 to fT4 ratio is associated with various markers of unfavorable metabolic profile and cardiovascular risk [45].

The existence of such a difference in prediction variability between boys and girls is rather interesting. Boys presented with higher prediction in all models regardless number of components. We assume that this might be due to the major influence of increased insulin level in girls as the consequence of pubertal physiological IR.

The major limitation of our study is the lack of data on the presence of acanthosis nigricans as the clinical sign of IR and the lack of the assessment of pubertal status in our adolescents. According to a unique longitudinal study performed in our country in years 1961–1982, the average age of pubertal maturity was 15.5–16.0 years in boys and 13.0 years in girls [46]. We thus assume that probably all of the girls and the majority of boys of our cohort have entered puberty at the time of investigation.

## Conclusions

In conclusion, type 2 diabetes in Czech adolescents even in obese individuals was rarely diagnosed. Findings of significantly higher level of blood glucose and higher prevalence of IFG regardless of weight status in boys than in girls lead us to a conclusion that adolescent boys in general are at greater risk of glucose disturbances in comparison to adolescent girls. This is further supported by our results that showed obese boys more insulin resistant than obese girls. In adolescence, HOMA-IR of 3.6 for girls and of 4.4 for boys were identified as cut-off values that corresponded to increased cardiometabolic risks. Several parameters seemed to play a role in HOMA-IR prediction. Major predictors for both genders were age, BMI, ratio of fT3 to fT4, GMT activity and levels of TG and SHBG.

## Abbreviations

BMI: Body mass index
FBG: Fasting blood glucose
fT3: Free triiodothyronine
fT4: Free thyroxine
GMT: Gamma-glutamyltransferase
HOMA-IR: Homeostasis model assessment of insulin resistance
IFG: Impaired fasting glucose
IR: Insulin resistance
OPLS: Orthogonal projections to latent structures
PAI-1: Plasminogen activator inhibitor-1
SHBG: Sex hormone-binding globulin
TG: Triglyceride
T2DM: Type 2 diabetes
US: United States.
